# Venturi Easy Ambient Ionization Mass Spectrometry Coupled to Gas Chromatography for High-Throughput Quantitation of Acetic Acid Residues in Pharmaceuticals

**DOI:** 10.1021/jasms.6c00025

**Published:** 2026-05-04

**Authors:** David Ulisses Tega, Luan Felipe Campos Oliveira, Thales Fernando Dias Pereira, Taynara Simão Matos, Patrick Cesar Ferreira, Heliara Dalva Lopes Nascimento, Marcos Nogueira Eberlin, Alessandra Sussulini

**Affiliations:** † Laboratory of Bioanalytics and Integrated Omics (LaBIOmics), Department of Analytical Chemistry, Institute of Chemistry, Universidade Estadual de Campinas (UNICAMP), 13083-970 Campinas, SP, Brazil; ‡ School of Engeneering and Mackgraphe, PPGEMN, 42524 Mackenzie Presbyterian University, 01302-907 São Paulo, SP, Brazil; § Instituto Nacional de Ciência e Tecnologia em Bioanalítica − Lauro Kubota (INCTBio-LK), Institute of Chemistry, Universidade Estadual de Campinas (UNICAMP), 13083-970 Campinas, SP, Brazil

**Keywords:** volatile organic compounds, gas chromatography, mass spectrometry, ambient ionization, V-EASI, pharmaceuticals

## Abstract

Gas chromatography coupled to mass spectrometry (GC-MS) is a robust and well-established technique for the analysis of volatile organic compounds (VOCs). Typically, electron ionization is used to combine these techniques; however, it requires high-vacuum conditions and produces extensive fragmentation. Venturi easy ambient sonic spray ionization (V-EASI) mass spectrometry is an ambient ionization approach suitable for samples in both solution and gas phases, employing high pressure as a soft ionization mechanism. In this work, we present a proof-of-principle for coupling GC to MS via V-EASI, applied to the quantification of acetic acid as a residual solvent in pharmaceutical products for quality control purposes. The system was automated and optimized, achieving a runtime of approximately 70 s per sample. The developed method meets the validation guidelines established by the International Council for Harmonization (ICH). Owing to its speed, simplicity, and broad applicability, GC-V-EASI-MS represents a powerful alternative for real-time, high-throughput VOC analysis.

## Graphical abstract

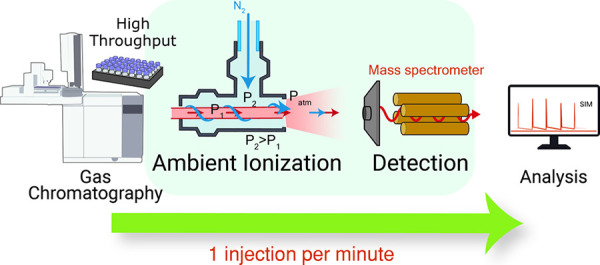

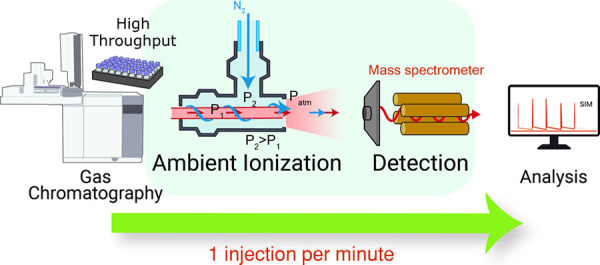

## Introduction

High-throughput mass spectrometry (HTMS) is a promising application of ambient ionization, enabling the rapid analysis of multiple samples and being primarily applied in drug discovery and bioassays.^1^ However, this approach could be valuable for pharmaceutical, food and beverage industries that require quality control of volatile organic compounds (VOCs) as routine analysis. The main ambient ionization alternatives that enable VOCs analysis in an HTMS approach are direct analysis in real time,^2^ desorption atmospheric pressure chemical ionization,^3^ low temperature plasma,^4^ and dielectric barrier discharge.^5^

Alternatively, Venturi easy ambient sonic spray ionization (V-EASI) is an ambient ionization technique that combines the Venturi effect with sonic spray ionization, enabling the direct analysis of samples in solution, in the gas phase, and on surfaces, without requiring electric fields, lasers, corona discharges, or any other kind of energy source for ion dissociation.^6^ V-EASI-MS has already been successfully applied in various fields, including natural products,^7^ fuels,^8,9^ lipids,^10^ and others complex matrices.^6^ Recently, we demonstrated its integration with liquid chromatography for the quantification of bixin in annatto seeds,^11^ and, within an HTMS strategy, coupled with online solid-phase extraction, for the quantification of caffeine in dietary supplements with an analysis time of 2 min per sample.^12^

In this work, a V-EASI-based interface operating as a HTMS strategy coupled with gas chromatography (GC) was applied to the quantification of acetic acid in pharmaceuticals. Acetic acid is an important chemical reagent widely used across various industrial sectors. In the pharmaceutical industry, it is extensively employed as a solvent and, consequently, may be present as a reaction residue.^13,14^ Due to its low toxic potential, the FDA (U.S. regulatory agency) classifies it as a Class 3 solvent, and the International Council for Harmonization guideline on residual solvents (ICH Q3C (R4)) establishes that acceptable levels must not exceed 5000 ppm.^15^ When this limit is exceeded, identification and quantification of the residue are required. Other common applications of acetic acid quantification include biomass valorization^16^ and the analysis of fermented foods.^17^ In this context, GC-V-EASI-MS is introduced for rapid analysis of acetic acid in complex matrices, requiring minimal sample preparation while delivering analytical performance comparable to or superior to conventional techniques.

## Experimental Section

### Chemicals

Acetonitrile, HPLC grade (Tedia, USA); acetic acid, ≥ 99% purity (Sigma-Aldrich, USA); ultrapure water (Milli-Q system, Millipore, USA); ammonium hydroxide, 29% (m/m) (Merck, USA); and capillary column with 100% polyethylene glycol stationary phase (DB-WAX) (Agilent, USA).

### Instrumentation

The GC-V-EASI-MS system assembled consisted of a three-concentric-tube interface (Figure 1 and Figure S1), built using a stainless-steel capillary (0.35 mm diameter) fixed concentrically between a needle (0.50 mm diameter) and a polyamide-coated fused-silica capillary (0.25 mm diameter). To attach the gas chromatograph to the ion source, a capillary segment with a length of 1.5 m, 0.25 mm inner diameter, and 0.25 μm film thickness was used. The intermediate line was connected to a modifier solution consisting of water/acetonitrile (1:1, v/v) containing 0.1% (m/v) ammonium hydroxide. This assembly enabled efficient deprotonation of acetic acid, thereby enhancing its ionization efficiency. The system was automated using an Arduino microcontroller programmed in C/C++, and two six-way pneumatically actuated valves, with stainless-steel and PEEK fittings.

**Figure 1:**
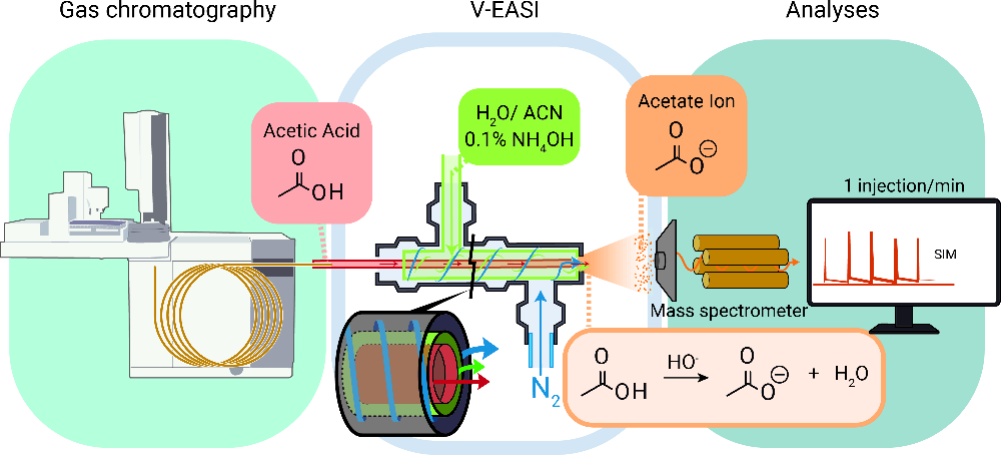
Representation of gas chromatography coupled to Venturi easy ambient sonic spray ionization mass spectrometry (GC-V-EASI-MS) for acetic acid analysis. ACN: acetonitrile, SIM: selected ion monitoring.

The GC system employed was a GC-17A (Shimadzu, Japan) coupled to a single-quadrupole mass spectrometer (LCMS-2010 EV, Shimadzu, Japan). Separation was performed using a DB-WAX column (2 m × 0.25 mm i.d., 0.25 μm film thickness), with helium as the carrier gas at a flow rate of 1 mL min^–1^. The oven temperature was held isothermally at 100 °C for 1 min. The mass analyzer was operated in negative ion mode, with a nebulizing gas flow rate of 5 L min^–1^, analyzer and block temperatures set to 250 °C, and a detector voltage of 1.40 kV. Detection was performed in selected ion monitoring (SIM) mode at *m*/*z* 59, corresponding to deprotonated acetic acid. To evaluate method accuracy relative to the official method, a conventional GC-MS system (5975C, Agilent, USA) was used.

### Methods

The ICH guideline for analytical method validation (Q2)^15^ was used. For the determination of acetic acid, an external calibration approach was employed. To evaluate precision, the relative standard deviation (RSD) was calculated, whereas accuracy was assessed by recovery (%), determined in agreement with the official method (conventional GC-MS). Rivaroxaban (a pharmaceutical used to prevent strokes) samples were used at different known concentrations, and a mixture of cellulose, croscarmellose, lactose, magnesium stearate, sodium lauryl sulfate, iron oxide, macrogol, and titanium dioxide was used as the excipient matrix. To perform the quantification of acetic acid as a residual solvent in rivaroxaban, solutions with a concentration range of 10–100 μg mL^–1^, prepared in Milli-Q water, were used. The modifier solution, 0.1% (m/v) ammonium hydroxide, was prepared in a 1:1 (v/v) water/acetonitrile mixture.

In the official method used for accuracy evaluation by GC-MS, the temperature program started at 100 °C and was held for 1 min, followed by a heating rate of 50 °C min^–1^ until 250 °C, which was maintained for 3 min, resulting in a total runtime of 5 min. The injector temperature was set to 280 °C, the transfer line was maintained at 280 °C, the ion source was operated at 280 °C, and the analyzer was set to 150 °C. Separation was performed using a capillary column composed of 5% diphenyl polysiloxane and 95% dimethyl polysiloxane, with a length of 30 m, 0.25 mm internal diameter, and 0.25 μm film thickness, operated at a constant flow rate of 1.0 mL min^–1^ and an injection volume of 1.0 μL.

## Results and Discussion

During acetic acid determination using the GC-V-EASI-MS system (Figure 1), the analyte was deprotonated in the gas phase after GC separation and prior to reaching the mass analyzer. Upon elution from the gas chromatographer, acetic acid interacted at the V-EASI interface with ammonium hydroxide, as illustrated in Figure 1. Deprotonation occurred within the concentric-tube interface, where a modifier solution consisting of water/acetonitrile (1:1, v/v) containing 0.1% (m/v) ammonium hydroxide in the microdroplet phase reacted with the analyte (acetic acid on gas phase), shifting the acid–base equilibrium and enabling quantitative detection by MS. Sample injection was performed using an automated system. In Figure 2, a sequence of 49 injections over 55 min is shown, corresponding to an average injection frequency of one analysis every 70 s. The injections performed up to 35 min correspond to standards at different concentration levels. In the time window between 38 and 55 min, the fortified sample used for the recovery test is presented. No peak broadening or saturation was observed, indicating no evidence of coelution, even when using low-resolution mass spectrometry.

**Figure 2:**
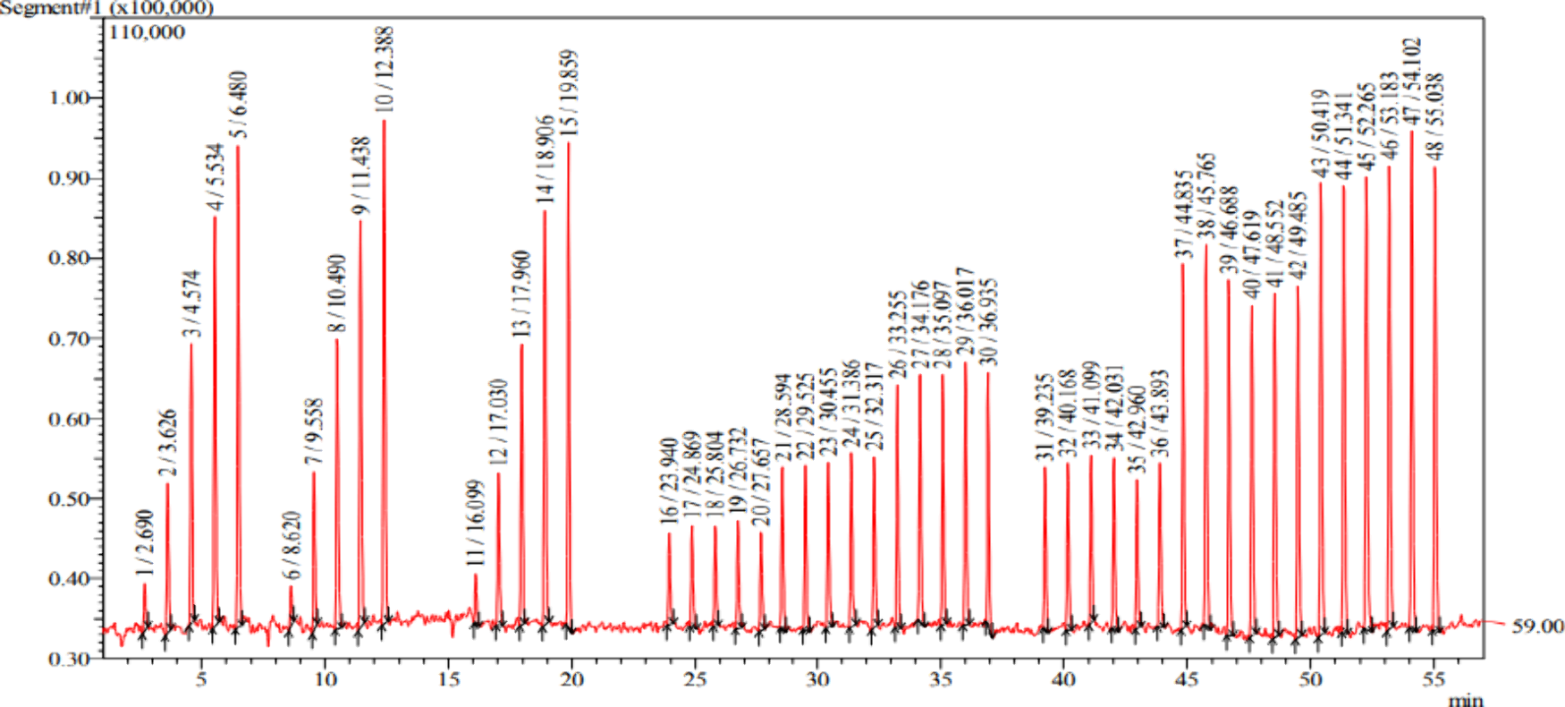
Chromatogram presenting successive and sequential injections of standards and samples, signals correspond to *m*/*z* 59 (acetate ions).

Linearity was evaluated to establish the working range for quantification using five calibration levels, each analyzed in triplicate, resulting in a total of 15 measurements. For the determination of acetic acid as a residual solvent in rivaroxaban by GC-V-EASI-MS, internal calibration was not required, and an external calibration approach was employed. This choice was justified by the use of GC coupling, which minimizes matrix effects and reduces ionization competition through effective sample separation. The working range determined for acetic acid in rivaroxaban was 10 to 100 μg mL^–1^, and the correlation coefficient (R) obtained for acetic acid quantification was 0.994. The residuals plot was also evaluated and is presented in Figure S2; a homogeneous dispersion of residuals was observed. The Cochran test was applied, and a homoscedastic calibration curve was obtained, with only random variance observed across the working range. These results comply with the validation criteria established by the ICH Q2 guidelines.^15^

Precision evaluates the dispersion of measurements within the working range of the developed methodology. It was assessed by calculating the relative standard deviation (RSD) of successive measurements performed on the same day (intraday precision) and on different days (interday precision) at three concentration levels (low, medium, and high). The results are presented in Table S1. Intraday RSD values ranged from 0.90 to 7.00%, and interday RSD values ranged from 1.40 to 5.60%. These results are in agreement with the ICH Q2 validation guidelines and with the Horwitz curve, which allows RSD values of up to 12.6% within the working range for acetic acid.^15^ Accuracy was expressed in terms of recovery, which measures the agreement between the values obtained by the official method and those obtained using the proposed methodology. The recovery results are presented in Table S2. Quantification of acetic acid in rivaroxaban using GC-V-EASI-MS yielded recovery values between 90.7% and 108.0%, which are consistent with the ICH Q2 validation guidelines.^15^

Using the GC-V-EASI-MS methodology, the analysis time was reduced to as little as 70 s per sample, representing, to the best of our knowledge, the fastest reported method for the quantification of acetic acid. For acetic acid as a residual solvent in rivaroxaban, previously reported methods typically require runtimes of approximately 20 min.^18^ The optimization of the analytical runtime did not compromise method performance, as both precision and accuracy remained in agreement with established validation guidelines. Specifically, quantification of acetic acid as a residual solvent in rivaroxaban using GC-V-EASI-MS yielded precision values in the range of 0.9 to 6.5% and recovery values of 101.2% ± 4.3%, whereas literature reports precision of 0.11%^18^ and recovery of 101.0% ± 0.7%.^17^ Thus, despite the substantial reduction in analysis time, the figures of merit were maintained and remain competitive with conventional approaches. Nevertheless, at present, GC-V-EASI-MS is limited to organic acid analytes due to its reliance on an online deprotonation step within the microdroplet phase following chromatographic separation, and further developments are required to expand the applicability of the system. Another limitation is the use of low resolution in SIM mode due to the employment of a single-quadrupole instrument, rather than high-resolution or selected reaction monitoring (SRM)-based approaches. This reduces the selectivity and specificity of the method, limiting its applicability to other matrices and requiring individual evaluation of each sample due to the potential for coelution.

## Conclusions

The concept of ionizing highly polar volatile compounds using V-EASI-MS coupled to gas chromatography was successfully demonstrated. Quantification of acetic acid by GC-V-EASI-MS achieved figures of merit consistent with the ICH Q2 validation guidelines. The automated interface developed in this study enabled optimization of the analytical runtime to approximately 1 min per sample, representing the shortest reported analysis time to date, while maintaining competitive analytical performance. This approach expands the ionization coverage of highly polar volatile compounds relevant to diverse scientific fields, including pharmaceuticals, food and beverages, environmental monitoring, health, microbiology, and renewable resources. Overall, this study addresses a key challenge in mass spectrometry by providing a soft, direct, and high-throughput strategy for the real-time analysis of polar VOCs at ambient pressure, thereby expanding the applicability of ambient ionization techniques to gas-phase separations. This is especially advantageous for the analysis of complex matrices, addressing one of the major challenges in mass spectrometry: matrix effects.

## Supplementary Material



## Notes

GC-V-EASI-MS instrumental setup (gas chromatography connection, NH_4_OH modifier line, and nitrogen gas line); residuals plot for acetic acid quantification in rivaroxaban, demonstrating the fit of the analytical method; method precision through relative standard deviation (RSD) values; recovery results, confirming the accuracy of the method (PDF)

## References

[1] Kulyk D. S., Amoah E., Badu-Tawiah A. K. (2020). High-Throughput Mass Spectrometry
Screening Platform for Discovering New Chemical Reactions under Uncatalyzed,
Solvent-Free Experimental Conditions. Anal.
Chem..

[2] Gross J. H. (2014). Direct
Analysis in Real Time—a Critical Review on DART-MS. Anal. Bioanal. Chem..

[3] Pitman C. N., LaCourse W. R. (2020). Desorption Atmospheric
Pressure Chemical Ionization:
A Review. Anal. Chim. Acta.

[4] Martínez-Jarquín S., Winkler R. (2017). Low-Temperature Plasma (LTP) Jets for Mass Spectrometry
(MS): Ion Processes, Instrumental Set-Ups, and Application Examples. TrAC Trends Anal. Chem..

[5] Guo C., Tang F., Chen J., Wang X., Zhang S., Zhang X. (2015). Development of Dielectric-Barrier-Discharge Ionization. Anal. Bioanal. Chem..

[6] Santos V. G., Regiani T., Dias F. F. G., Romão W., Jara J. L. P., Klitzke C. F., Coelho F., Eberlin M. N. (2011). Venturi
Easy Ambient Sonic-Spray Ionization. Anal. Chem..

[7] Cabral E. C., Simas R. C., Santos V. G., Queiroga C. L., da Cunha V. S., de Sá G. F., Daroda R. J., Eberlin M. N. (2012). Wood Typification
by Venturi Easy Ambient Sonic Spray Ionization Mass Spectrometry:
The Case of the Endangered Mahogany Tree. J.
Mass Spectrom..

[8] Haddad R., Regiani T., Klitzke C. F., Sanvido G. B., Corilo Y. E., Augusti D. V., Pasa V. M. D., Pereira R. C. C., Romão W., Vaz B. G., Augusti R., Eberlin M. N. (2012). Gasoline, Kerosene,
and Diesel Fingerprinting via Polar Markers. Energy Fuels.

[9] Tega D. U., Nascimento H., Jara J. L., Santos J. M., Eberlin M. N. (2020). A Rapid
and Versatile Method to Determine Methanol in Biofuels and Gasoline
by Ambient Mass Spectrometry Using a V-EASI Source. Energy Fuels.

[10] Schäfer K.-C., Balog J., Szaniszló T., Szalay D., Mezey G., Dénes J., Bognár L., Oertel M., Takáts Z. (2011). Real Time
Analysis of Brain Tissue by Direct Combination of Ultrasonic Surgical
Aspiration and Sonic Spray Mass Spectrometry. Anal. Chem..

[11] Oliveira L. F. C., Tega D. U., Eberlin M. N., Sussulini A. (2022). Liquid Chromatography
Coupled to Venturi Easy Ambient Sonic Spray Ionization Mass Spectrometry. Talanta.

[12] Tega D. U., Campos Oliveira L. F., Ferreira P. C., Soldera B. B., Nascimento H. D. L., Eberlin M. N., Sussulini A. (2024). Caffeine Quantification
in Dietary
Supplements Using High-Throughput on-Line Solid Phase Extraction Coupled
to Venturi Easy Ambient Sonic-Spray Ionization Mass Spectrometry. Anal. Meth..

[13] Grodowska K., Parczewski A. (2010). Organic Solvents
in the Pharmaceutical Industry. Acta Polym.
Pharm..

[14] Reid, G. L. Chapter 17 - Residual Solvents. In Specification of Drug Substances and Products, Third ed.; Riley, C. M. ; Nguyen, K. L. , Eds.; Elsevier, 2025; pp 421–438. 10.1016/B978-0-443-13466-1.00036-2.

[15] International Council for Harmonisation (ICH) . ICH Q2(R2) Guideline on Validation of Analytical Procedures, 2022; Step 5.

[16] Humpula J.
F., Chundawat S. P. S., Vismeh R., Jones A. D., Balan V., Dale B. E. (2011). Rapid Quantification
of Major Reaction Products Formed
during Thermochemical Pretreatment of Lignocellulosic Biomass Using
GC–MS. J. Chromatogr. B.

[17] Pinu F., Villas-boas S. G. (2017). Rapid Quantification of Major Volatile
Metabolites
in Fermented Food and Beverages Using Gas Chromatography-Mass Spectrometry. Metabolites.

[18] Poronsky C. J., Cutrone J. Q. (2017). Chromatoprobe as a Sample-Sparing Technique for Residual
Solvent Analysis of Drug Discovery Candidates by Gas Chromatography. J. Pharm. Analysis.

