# Supplementary material for: Venturi Easy Ambient Ionization Mass Spectrometry Coupled to Gas Chromatography for High-Throughput Quantitation of Acetic Acid Residues in Pharmaceuticals
Source: J Am Soc Mass Spectrom. 2026 May 4;37(6):1321–4. doi: 10.1021/jasms.6c00025 (PMC13237769; doi:10.1021/jasms.6c00025)
Supplement: Supplementary file 1 [file js6c00025_si_001.pdf]

Supporting Information

Venturi Easy Ambient Ionization Mass Spectrometry Coupled to Gas Chromatography for High-Throughput Quantitation of Acetic Acid Residues in Pharmaceuticals

David Ulisses Tega[a,†], Luan Felipe Campos Oliveira[a,†], Thales Fernando Dias Pereira[a], Taynara Simão Matos[a], Patrick Cesar Ferreira[a], Heliara Dalva Lopes Nascimento[a], Marcos Nogueira Eberlin[b], and Alessandra Sussulini[a,c,*]

[a] *Laboratory of Bioanalytics and Integrated Omics (LaBIOmics), Department of Analytical Chemistry, Institute of Chemistry, Universidade Estadual de Campinas (UNICAMP), 13083-970, Campinas, SP, Brazil.*

[b] *School of Engeneering and Mackgraphe, PPGEMN, Mackenzie Presbyterian University, 01302-907, São Paulo, SP, Brazil.*

[c] *Instituto Nacional de Ciência e Tecnologia em Bioanalítica – Lauro Kubota (INCTBio-LK), Institute of Chemistry, Universidade Estadual de Campinas (UNICAMP), 13083-970, Campinas, SP, Brazil.*

**Corresponding author*

sussulini@unicamp.br

[†] *These authors contributed equally.*

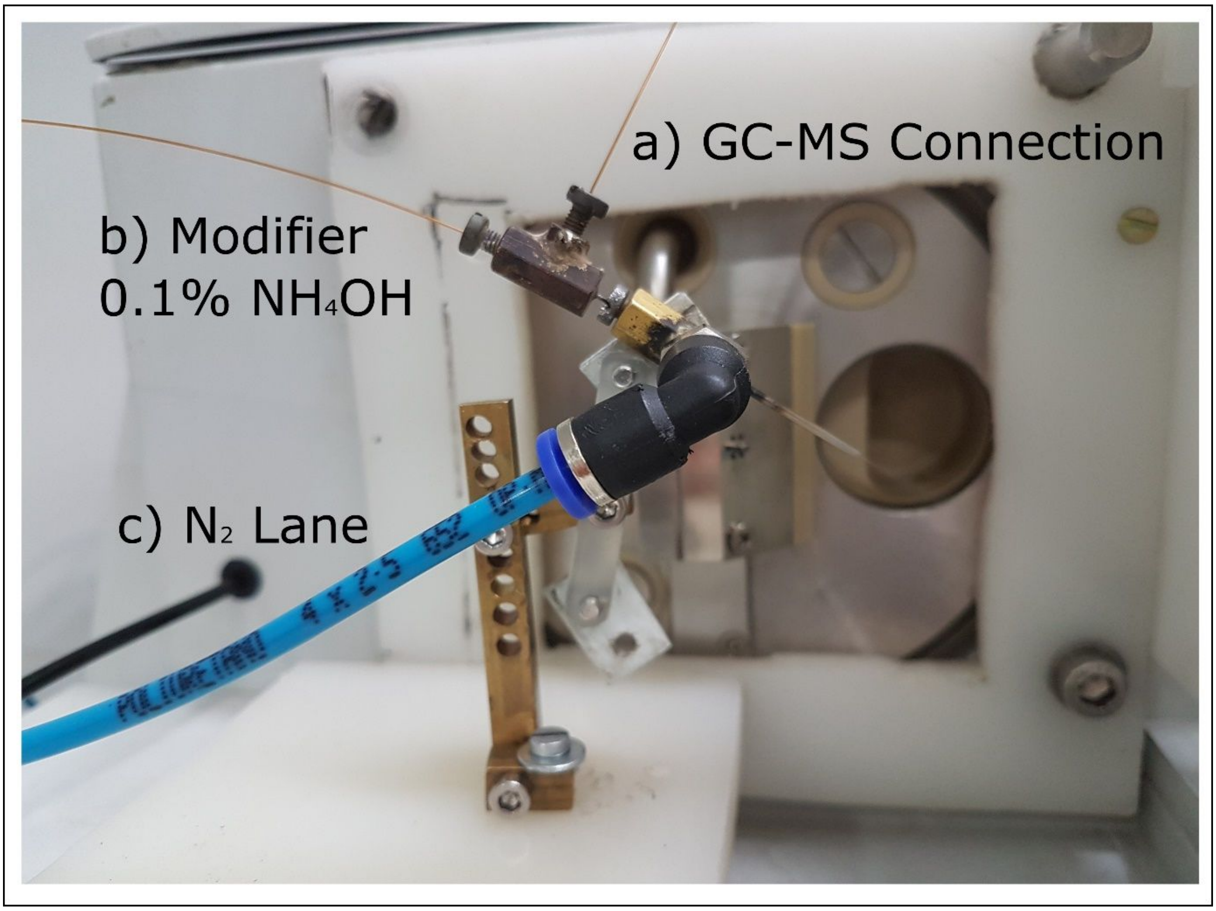

Figure S1. GC-V-EASI-MS system: a) gas chromatography connection, b) modifier 0.1% (m/v) NH_4OH connection, c) nitrogen lane connection.

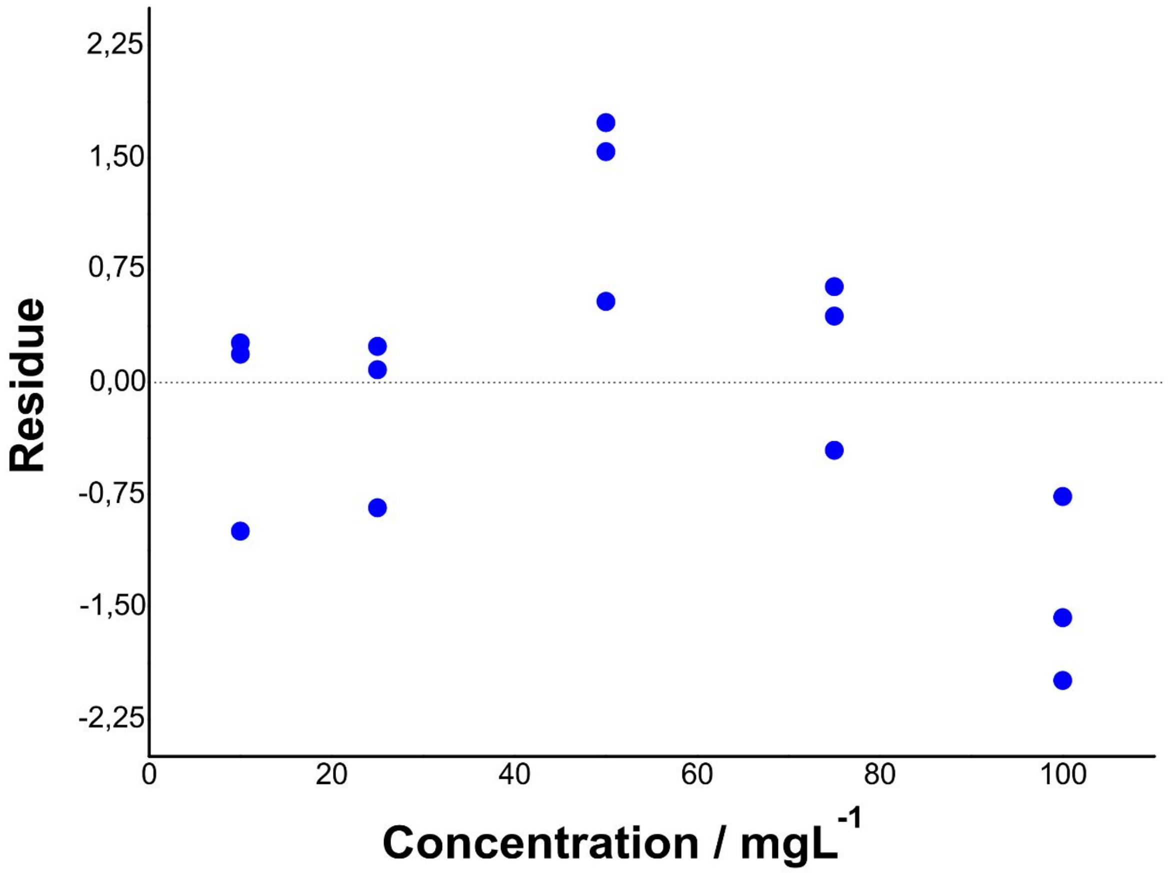

Figure S2. Residuals plot for the quantification of acetic acid in rivaboraxan using GC-V-EASI-MS.

Table S1. Relative standard deviation (RSD) for the quantification acetic acid in rivaboraxan using GC-V-EASI-MS

Concentration (mg kg^{-1})	RSD (%)			
	Day 1 (n=5)	Day 2 (n=5)	Day 3 (n=5)	Intermediate Precision (n=15)
885	5.70	5.50	6.50	5.60
1460	0.90	7.00	1.50	1.40
2085	5.90	1.60	2.80	2.70

Table S2. Recovery results for the quantification of acetic acid in rivaboraxan via GC-V-EASI-MS

Concentration (mg kg^{-1})	Recovery (%)		
	Day 1 (n=5)	Day 2 (n=5)	Day 3 (n=5)
885	90.7	106.0	104.0
1460	99.7	105.0	99.1
2085	108.0	101.0	97.6
